# {2-[(Benzo­yloxy)meth­yl]-1-oxo-3*H*-pyrrolizin-2-yl}methyl benzoate

**DOI:** 10.1107/S1600536810051974

**Published:** 2010-12-18

**Authors:** Yousaf Ali, Sammer Yousuf, Nighat Afza, Yu Peng, Mahboob Ali Kalhoro

**Affiliations:** a Pharmaceutical Research Center, PCSIR Laboratories Complex, Karachi 75280, Pakistan; b H.E.J. Research Institute of Chemistry, International Center for Chemical and Biological Sciences, University of Karachi, Karachi 75270, Pakistan; c Department of Pharmaceutical Engineering, Biotechnology College, Tianjin University of Science & Technology, Tianjin 300457, People’s Republic of China

## Abstract

The title compound, C_23_H_19_NO_5_, was prepared by esterification of 2,2-bis­(hy­droxy­meth­yl)-2,3-dihydro-1*H*-pyrrolizin-1-one with benzoyl chloride in pyridine·The pyrrolizine ring system is approximately planar with a maximum deviation of 0.008 (2) Å from the least-squares plane; the two phenyl rings are oriented at dihedral angles of 64.26 (11) and 70.75 (10)° with respect to the pyrrolizine ring system. Weak inter­molecular C—H⋯O hydrogen bonding occurs in the crystal structure.

## Related literature

For general background to 2,3-dihydro­pyrrolizine derivatives and for the biological activity of related compounds, see: Skvortsov & Astakhova (1992 ▶); Albrecht *et al.* (2008 ▶); Morúaa *et al.* (2009 ▶). For side effects of non-steroidal anti-inflammatory drugs, see: Mishra *et al.* (2008 ▶). For the synthesis, see: Clemo & Ramage (1931 ▶). For the natural source of the compound, see: Meinwald & Meinwald (1965 ▶). For related structures, see: Ali *et al.* (2010*a* ▶,*b* ▶,*c* ▶).
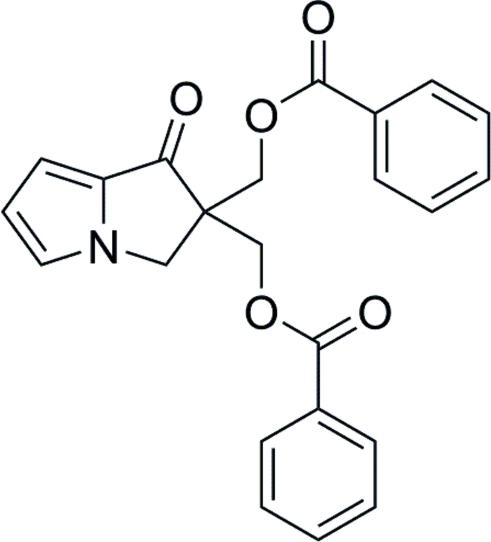


## Experimental

### 

#### Crystal data

C_23_H_19_NO_5_
*M* *_r_* = 389.39
Triclinic, 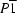

*a* = 8.0438 (8) Å
*b* = 11.9359 (13) Å
*c* = 12.0614 (13) Å
α = 64.417 (2)°
β = 72.670 (2)°
γ = 77.390 (2)°
*V* = 991.65 (18) Å^3^
*Z* = 2
Mo *K*α radiation
μ = 0.09 mm^−1^
*T* = 298 K
0.42 × 0.20 × 0.14 mm

#### Data collection

Bruker SMART APEX CCD area-detector diffractometer
13906 measured reflections
4932 independent reflections
3206 reflections with *I* > 2σ(*I*)
*R*_int_ = 0.026

#### Refinement

*R*[*F* ^2^ > 2σ(*F* ^2^)] = 0.057
*wR*(*F* ^2^) = 0.161
*S* = 1.01
4932 reflections
262 parameters
H-atom parameters constrained
Δρ_max_ = 0.46 e Å^−3^
Δρ_min_ = −0.22 e Å^−3^

### 

Data collection: *SMART* (Bruker, 2000 ▶); cell refinement: *SAINT* (Bruker, 2000 ▶); data reduction: *SAINT*; program(s) used to solve structure: *SHELXS97* (Sheldrick, 2008 ▶); program(s) used to refine structure: *SHELXL97* (Sheldrick, 2008 ▶); molecular graphics: *ORTEP-3* (Farrugia, 1997 ▶); software used to prepare material for publication: *publCIF* (Westrip, 2010 ▶) and *PLATON* (Spek, 2009) ▶.

## Supplementary Material

Crystal structure: contains datablocks I, global. DOI: 10.1107/S1600536810051974/xu5119sup1.cif

Structure factors: contains datablocks I. DOI: 10.1107/S1600536810051974/xu5119Isup2.hkl

Additional supplementary materials:  crystallographic information; 3D view; checkCIF report

## Figures and Tables

**Table 1:** Hydrogen-bond geometry (Å, °)

*D*—H⋯*A*	*D*—H	H⋯*A*	*D*⋯*A*	*D*—H⋯*A*
C4—H4*A*⋯O3^i^	0.93	2.59	3.299 (3)	133
C20—H20*A*⋯O3^ii^	0.93	2.44	3.269 (3)	149

Symmetry codes: (i) 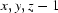; (ii) 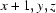.

**Fig. 1.**
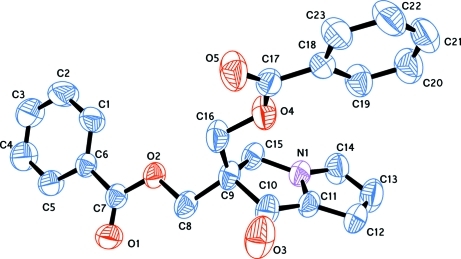
View of the single molecule showing atom numbering scheme at 50% ellipsoids probability level. Hydrogen atoms are Omitted for clarity.

**Fig. 2.**
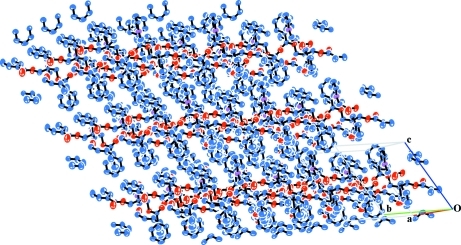
Packing diagram showing infinite chains parallel to c axis.

**Fig. 3.**
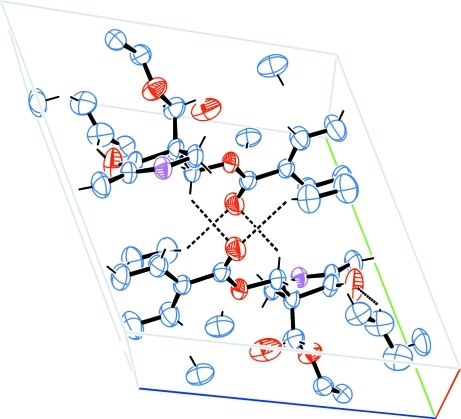
Veiw of cell unit showing Hydrogen bonding as dashed lines.

**Fig. 4.**
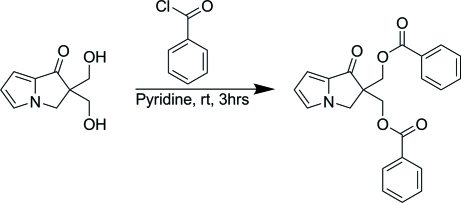
Chemical Reaction Scheme.

## supplementary crystallographic information

### Comment

Derivatives of 2,3-dihydropyrrolizine became known through studies of their synthesis (Clemo & Ramage, 1931) and isolation from natural source (Meinwald & Meinwald, 1965). Depending on their structure, derivatives of 2,3-dihydropyrrolizine have shown merit as analgesics, anti-inflammatory agents, myorelaxants, inhibitors of thrombocyte aggregation, fibrinolytics, temperature-lowering substances and drugs for the treatment of glaucoma and conjunctivitis (Skvortsov, 1992). The most important of these, Ketorolac, is reported in literature as one of the most effective nonsteroidal anti-inflammatory drugs to alleviate renoureteral colic (Morúaa *et al.*, 2009). But it suffers from the general side effects of NSAIDs, owing to presence of free carboxylic acid group (Mishra *et al.*, 2008). Licofelone(2-[6-(4-Chlorophenyl)-2,2-dimethyl-7-phenyl-2,3-dihydro- 1Hpyrrolizin-5-yl] acetic acid) is a dual inhibitor of both cyclooxygenase isoforms and 5-lipoxygenase (Albrecht *et al.*, 2008). Title compound was prepared in order to synthesize new derivatives of this series. Crystal structures of related molecules are reported (Ali *et al.*, 2010a,b,c).

Numbering scheme for single molecule of the title compound is shown in an *ORTEP* (Farrugia, 1997) plot of the molecule at 50% ellipsoid probability limit (Fig. 1). The two phenyl rings (C1—C6 and C18—C23) and central pyrollizine ring (C9—C15) are each planner with maximum deviation of 0.006 (3)Å for C5, 0.007 (2)Å for C23 and 0.008 (2)Å for C9 atom from the least square planes, respectively. In the crystal structure, the molecules are stabilized, to form a two-dimensional network or infinite chains along *z* axis (Fig.2), by intermolecular hydrogen bonds C—H···O (Fig.3, symmetry codes as in Table 1).

### Experimental

Title compound was prepared by esterification of 2,2-bis(hydroxymethyl)-2,3-dihydro-1*H*-pyrrolizin- 1-one (1) with benzylchloride (2) (Fig. 4). Thus a mixture of one mole percent of 1 and 1.1 mole percent of 2 was stirred in pyridine at room temperature for three hours. The product was precipitated out by addition of cold water and filtered out to give title compound in good yeild. Final product was purified by Flash Colum Chromatography (Ethyl Acetate: Petroleum Ether = 1:1). Single crystals for X-ray analysis were grown by evaporation from a dilute solution in Ethyl Acetate: Petroleum Ether = 1:1.

### Refinement

All H atoms were placed in geometrically idealized positions and constrained to ride on their parent atoms with C—H = 0.93 and 0.97 Å for aromatic and methylene, respectively. *U*_iso_(H) values were taken to be equal to 1.2 *U*_eq_(C) for all hydrogen atoms.

### Crystal data

**Table:**

C_23_H_19_NO_5_	*Z* = 2
*M**_r_* = 389.39	*F*(000) = 408
Triclinic, *P*1	*D*_x_ = 1.304 Mg m^−^^3^
Hall symbol: -P 1	Mo *K*α radiation, λ = 0.71073 Å
*a* = 8.0438 (8) Å	Cell parameters from 4932 reflections
*b* = 11.9359 (13) Å	θ = 1.9–28.3°
*c* = 12.0614 (13) Å	µ = 0.09 mm^−^^1^
α = 64.417 (2)°	*T* = 298 K
β = 72.670 (2)°	Block, colourless
γ = 77.390 (2)°	0.42 × 0.20 × 0.14 mm
*V* = 991.65 (18) Å^3^	

### Data collection

**Table:**

Bruker SMART APEX CCD area-detector diffractometer	3206 reflections with *I* > 2σ(*I*)
Radiation source: fine-focus sealed tube	*R*_int_ = 0.026
graphite	θ_max_ = 28.3°, θ_min_ = 1.9°
Detector resolution: 83.66 pixels mm^-1^	*h* = −10→10
ω scans	*k* = −15→15
13906 measured reflections	*l* = −16→16
4932 independent reflections	

### Refinement

**Table:**

Refinement on *F*^2^	Primary atom site location: structure-invariant direct methods
Least-squares matrix: full	Secondary atom site location: difference Fourier map
*R*[*F*^2^ > 2σ(*F*^2^)] = 0.057	Hydrogen site location: inferred from neighbouring sites
*wR*(*F*^2^) = 0.161	H-atom parameters constrained
*S* = 1.01	*w* = 1/[σ^2^(*F*_o_^2^) + (0.0698*P*)^2^ + 0.1946*P*] where *P* = (*F*_o_^2^ + 2*F*_c_^2^)/3
4932 reflections	(Δ/σ)_max_ < 0.001
262 parameters	Δρ_max_ = 0.46 e Å^−^^3^
0 restraints	Δρ_min_ = −0.22 e Å^−^^3^

### Special details

**Table:**

Geometry. All e.s.d.'s (except the e.s.d. in the dihedral angle between two l.s. planes) are estimated using the full covariance matrix. The cell e.s.d.'s are taken into account individually in the estimation of e.s.d.'s in distances, angles and torsion angles; correlations between e.s.d.'s in cell parameters are only used when they are defined by crystal symmetry. An approximate (isotropic) treatment of cell e.s.d.'s is used for estimating e.s.d.'s involving l.s. planes.
Refinement. Refinement of *F*^2^ against ALL reflections. The weighted *R*-factor *wR* and goodness of fit *S* are based on *F*^2^, conventional *R*-factors *R* are based on *F*, with *F* set to zero for negative *F*^2^. The threshold expression of *F*^2^ > σ(*F*^2^) is used only for calculating *R*-factors(gt) *etc*. and is not relevant to the choice of reflections for refinement. *R*-factors based on *F*^2^ are statistically about twice as large as those based on *F*, and *R*- factors based on ALL data will be even larger.

### Fractional atomic coordinates and isotropic or equivalent isotropic displacement parameters (Å^2^)

**Table:**

	*x*	*y*	*z*	*U*_iso_*/*U*_eq_	
O1	0.3231 (2)	0.62080 (14)	0.45737 (13)	0.0694 (4)	
O2	0.55937 (16)	0.70317 (12)	0.43879 (11)	0.0533 (3)	
O3	0.4749 (2)	0.66084 (19)	0.82292 (15)	0.0944 (6)	
O4	0.7843 (2)	0.85334 (13)	0.61521 (14)	0.0689 (4)	
O5	0.6833 (2)	1.05268 (15)	0.57098 (18)	0.0907 (6)	
N1	0.8947 (2)	0.56213 (15)	0.69252 (13)	0.0537 (4)	
C1	0.6217 (3)	0.8044 (2)	0.17996 (19)	0.0714 (6)	
H1A	0.6835	0.8284	0.2187	0.086*	
C2	0.6589 (4)	0.8481 (3)	0.0492 (2)	0.0926 (9)	
H2A	0.7452	0.9019	0.0003	0.111*	
C3	0.5692 (4)	0.8123 (3)	−0.0073 (2)	0.0869 (8)	
H3A	0.5949	0.8415	−0.0950	0.104*	
C4	0.4434 (4)	0.7349 (2)	0.0627 (2)	0.0818 (7)	
H4A	0.3832	0.7105	0.0231	0.098*	
C5	0.4036 (3)	0.6918 (2)	0.19253 (19)	0.0668 (6)	
H5A	0.3154	0.6395	0.2404	0.080*	
C6	0.4945 (2)	0.72617 (16)	0.25171 (16)	0.0479 (4)	
C7	0.4475 (2)	0.67765 (16)	0.39199 (16)	0.0486 (4)	
C8	0.5139 (3)	0.66392 (18)	0.57460 (16)	0.0559 (5)	
H8A	0.5044	0.5748	0.6154	0.067*	
H8B	0.4021	0.7070	0.6007	0.067*	
C9	0.6569 (3)	0.69529 (17)	0.61193 (16)	0.0531 (5)	
C10	0.6152 (3)	0.63955 (19)	0.75869 (17)	0.0575 (5)	
C11	0.7683 (2)	0.56484 (17)	0.79665 (16)	0.0513 (4)	
C12	0.8376 (3)	0.4943 (2)	0.90126 (19)	0.0686 (6)	
H12A	0.7816	0.4800	0.9850	0.082*	
C13	1.0058 (3)	0.4495 (3)	0.8577 (2)	0.0821 (7)	
H13A	1.0838	0.3989	0.9075	0.098*	
C14	1.0393 (3)	0.4921 (2)	0.7279 (2)	0.0730 (6)	
H14A	1.1429	0.4753	0.6747	0.088*	
C15	0.8401 (3)	0.63095 (18)	0.57321 (16)	0.0562 (5)	
H15A	0.8340	0.5748	0.5360	0.067*	
H15B	0.9201	0.6920	0.5134	0.067*	
C16	0.6544 (3)	0.83585 (18)	0.5645 (2)	0.0647 (5)	
H16A	0.6843	0.8735	0.4729	0.078*	
H16B	0.5396	0.8729	0.5947	0.078*	
C17	0.7803 (3)	0.96426 (18)	0.61761 (18)	0.0553 (5)	
C18	0.9055 (2)	0.96183 (17)	0.68757 (17)	0.0520 (4)	
C19	1.0125 (3)	0.8561 (2)	0.7402 (2)	0.0678 (6)	
H19A	1.0122	0.7839	0.7288	0.081*	
C20	1.1203 (3)	0.8573 (2)	0.8099 (3)	0.0820 (7)	
H20A	1.1929	0.7858	0.8449	0.098*	
C21	1.1210 (3)	0.9627 (3)	0.8276 (3)	0.0848 (7)	
H21A	1.1931	0.9627	0.8753	0.102*	
C22	1.0158 (3)	1.0683 (2)	0.7753 (3)	0.0856 (8)	
H22A	1.0161	1.1400	0.7875	0.103*	
C23	0.9094 (3)	1.0683 (2)	0.7047 (2)	0.0704 (6)	
H23A	0.8395	1.1408	0.6681	0.084*	

### Atomic displacement parameters (Å^2^)

**Table:**

	*U*^11^	*U*^22^	*U*^33^	*U*^12^	*U*^13^	*U*^23^
O1	0.0775 (10)	0.0854 (10)	0.0497 (8)	−0.0319 (8)	−0.0126 (7)	−0.0209 (7)
O2	0.0670 (8)	0.0615 (8)	0.0392 (6)	−0.0112 (6)	−0.0191 (6)	−0.0204 (6)
O3	0.0874 (12)	0.1345 (16)	0.0621 (10)	0.0270 (11)	−0.0207 (9)	−0.0554 (10)
O4	0.0845 (10)	0.0583 (8)	0.0831 (10)	0.0067 (7)	−0.0485 (8)	−0.0329 (7)
O5	0.1067 (13)	0.0594 (9)	0.1268 (15)	0.0124 (9)	−0.0780 (12)	−0.0321 (9)
N1	0.0598 (9)	0.0614 (10)	0.0416 (8)	−0.0062 (7)	−0.0165 (7)	−0.0187 (7)
C1	0.0742 (14)	0.0969 (16)	0.0524 (11)	−0.0331 (12)	−0.0164 (10)	−0.0254 (11)
C2	0.0941 (18)	0.127 (2)	0.0526 (13)	−0.0546 (17)	−0.0050 (12)	−0.0194 (14)
C3	0.1055 (19)	0.118 (2)	0.0402 (11)	−0.0312 (16)	−0.0152 (12)	−0.0256 (12)
C4	0.1089 (19)	0.1024 (19)	0.0540 (12)	−0.0312 (15)	−0.0293 (13)	−0.0326 (12)
C5	0.0874 (15)	0.0748 (14)	0.0505 (11)	−0.0283 (11)	−0.0223 (10)	−0.0225 (10)
C6	0.0548 (10)	0.0505 (10)	0.0432 (9)	−0.0038 (8)	−0.0173 (8)	−0.0197 (8)
C7	0.0577 (11)	0.0498 (10)	0.0446 (9)	−0.0045 (8)	−0.0180 (8)	−0.0206 (8)
C8	0.0724 (12)	0.0609 (11)	0.0391 (9)	−0.0075 (9)	−0.0183 (9)	−0.0202 (8)
C9	0.0686 (12)	0.0568 (11)	0.0415 (9)	0.0010 (9)	−0.0231 (8)	−0.0232 (8)
C10	0.0684 (12)	0.0700 (13)	0.0429 (10)	0.0037 (10)	−0.0178 (9)	−0.0321 (9)
C11	0.0632 (11)	0.0585 (11)	0.0363 (9)	−0.0074 (9)	−0.0155 (8)	−0.0195 (8)
C12	0.0811 (15)	0.0813 (15)	0.0422 (10)	−0.0107 (12)	−0.0227 (10)	−0.0166 (10)
C13	0.0791 (16)	0.0966 (18)	0.0647 (14)	0.0073 (13)	−0.0366 (12)	−0.0211 (13)
C14	0.0586 (12)	0.0927 (17)	0.0661 (13)	0.0062 (11)	−0.0233 (10)	−0.0306 (12)
C15	0.0709 (12)	0.0633 (12)	0.0392 (9)	−0.0046 (9)	−0.0170 (9)	−0.0229 (8)
C16	0.0837 (14)	0.0601 (12)	0.0653 (12)	0.0008 (10)	−0.0422 (11)	−0.0260 (10)
C17	0.0616 (11)	0.0504 (11)	0.0547 (11)	−0.0034 (9)	−0.0213 (9)	−0.0176 (9)
C18	0.0478 (10)	0.0543 (11)	0.0521 (10)	−0.0074 (8)	−0.0132 (8)	−0.0171 (8)
C19	0.0672 (13)	0.0619 (13)	0.0826 (15)	0.0046 (10)	−0.0339 (11)	−0.0304 (11)
C20	0.0737 (15)	0.0752 (15)	0.1005 (19)	0.0050 (12)	−0.0476 (14)	−0.0259 (14)
C21	0.0734 (15)	0.0923 (18)	0.1029 (19)	−0.0155 (13)	−0.0445 (14)	−0.0326 (15)
C22	0.0815 (16)	0.0752 (16)	0.123 (2)	−0.0121 (12)	−0.0450 (16)	−0.0446 (15)
C23	0.0637 (13)	0.0565 (12)	0.0986 (17)	−0.0037 (9)	−0.0348 (12)	−0.0283 (12)

### Geometric parameters (Å, °)

**Table:**

O1—C7	1.203 (2)	C9—C15	1.544 (3)
O2—C7	1.339 (2)	C9—C10	1.554 (2)
O2—C8	1.446 (2)	C10—C11	1.423 (3)
O3—C10	1.209 (2)	C11—C12	1.382 (2)
O4—C17	1.330 (2)	C12—C13	1.377 (3)
O4—C16	1.448 (2)	C12—H12A	0.9300
O5—C17	1.195 (2)	C13—C14	1.380 (3)
N1—C14	1.340 (2)	C13—H13A	0.9300
N1—C11	1.368 (2)	C14—H14A	0.9300
N1—C15	1.462 (2)	C15—H15A	0.9700
C1—C6	1.364 (3)	C15—H15B	0.9700
C1—C2	1.387 (3)	C16—H16A	0.9700
C1—H1A	0.9300	C16—H16B	0.9700
C2—C3	1.359 (3)	C17—C18	1.483 (3)
C2—H2A	0.9300	C18—C19	1.377 (3)
C3—C4	1.348 (3)	C18—C23	1.381 (3)
C3—H3A	0.9300	C19—C20	1.381 (3)
C4—C5	1.378 (3)	C19—H19A	0.9300
C4—H4A	0.9300	C20—C21	1.366 (4)
C5—C6	1.380 (2)	C20—H20A	0.9300
C5—H5A	0.9300	C21—C22	1.367 (3)
C6—C7	1.486 (2)	C21—H21A	0.9300
C8—C9	1.520 (3)	C22—C23	1.375 (3)
C8—H8A	0.9700	C22—H22A	0.9300
C8—H8B	0.9700	C23—H23A	0.9300
C9—C16	1.518 (3)		
			
C7—O2—C8	114.90 (14)	C12—C11—C10	142.85 (19)
C17—O4—C16	118.43 (15)	C13—C12—C11	106.76 (19)
C14—N1—C11	109.83 (16)	C13—C12—H12A	126.6
C14—N1—C15	135.85 (17)	C11—C12—H12A	126.6
C11—N1—C15	114.29 (15)	C12—C13—C14	108.64 (19)
C6—C1—C2	120.1 (2)	C12—C13—H13A	125.7
C6—C1—H1A	120.0	C14—C13—H13A	125.7
C2—C1—H1A	120.0	N1—C14—C13	107.3 (2)
C3—C2—C1	119.9 (2)	N1—C14—H14A	126.4
C3—C2—H2A	120.0	C13—C14—H14A	126.4
C1—C2—H2A	120.0	N1—C15—C9	103.49 (14)
C4—C3—C2	120.5 (2)	N1—C15—H15A	111.1
C4—C3—H3A	119.7	C9—C15—H15A	111.1
C2—C3—H3A	119.7	N1—C15—H15B	111.1
C3—C4—C5	120.2 (2)	C9—C15—H15B	111.1
C3—C4—H4A	119.9	H15A—C15—H15B	109.0
C5—C4—H4A	119.9	O4—C16—C9	105.05 (14)
C4—C5—C6	120.1 (2)	O4—C16—H16A	110.7
C4—C5—H5A	119.9	C9—C16—H16A	110.7
C6—C5—H5A	119.9	O4—C16—H16B	110.7
C1—C6—C5	119.15 (17)	C9—C16—H16B	110.7
C1—C6—C7	122.40 (16)	H16A—C16—H16B	108.8
C5—C6—C7	118.44 (17)	O5—C17—O4	122.90 (18)
O1—C7—O2	123.14 (16)	O5—C17—C18	125.42 (18)
O1—C7—C6	124.05 (16)	O4—C17—C18	111.66 (16)
O2—C7—C6	112.81 (15)	C19—C18—C23	118.99 (19)
O2—C8—C9	108.01 (15)	C19—C18—C17	122.41 (18)
O2—C8—H8A	110.1	C23—C18—C17	118.54 (17)
C9—C8—H8A	110.1	C18—C19—C20	120.0 (2)
O2—C8—H8B	110.1	C18—C19—H19A	120.0
C9—C8—H8B	110.1	C20—C19—H19A	120.0
H8A—C8—H8B	108.4	C21—C20—C19	120.4 (2)
C16—C9—C8	110.34 (15)	C21—C20—H20A	119.8
C16—C9—C15	112.71 (17)	C19—C20—H20A	119.8
C8—C9—C15	113.55 (15)	C20—C21—C22	120.0 (2)
C16—C9—C10	108.49 (15)	C20—C21—H21A	120.0
C8—C9—C10	106.88 (16)	C22—C21—H21A	120.0
C15—C9—C10	104.41 (14)	C21—C22—C23	120.0 (2)
O3—C10—C11	129.35 (18)	C21—C22—H22A	120.0
O3—C10—C9	123.08 (18)	C23—C22—H22A	120.0
C11—C10—C9	107.57 (16)	C22—C23—C18	120.6 (2)
N1—C11—C12	107.50 (17)	C22—C23—H23A	119.7
N1—C11—C10	109.64 (15)	C18—C23—H23A	119.7
			
C6—C1—C2—C3	−0.4 (4)	C9—C10—C11—C12	175.6 (3)
C1—C2—C3—C4	0.3 (5)	N1—C11—C12—C13	−0.4 (2)
C2—C3—C4—C5	0.4 (4)	C10—C11—C12—C13	−179.3 (3)
C3—C4—C5—C6	−1.0 (4)	C11—C12—C13—C14	0.1 (3)
C2—C1—C6—C5	−0.2 (4)	C11—N1—C14—C13	−0.5 (3)
C2—C1—C6—C7	−179.2 (2)	C15—N1—C14—C13	−178.2 (2)
C4—C5—C6—C1	0.9 (3)	C12—C13—C14—N1	0.2 (3)
C4—C5—C6—C7	180.0 (2)	C14—N1—C15—C9	−176.2 (2)
C8—O2—C7—O1	−3.6 (3)	C11—N1—C15—C9	6.1 (2)
C8—O2—C7—C6	176.72 (14)	C16—C9—C15—N1	110.08 (16)
C1—C6—C7—O1	170.9 (2)	C8—C9—C15—N1	−123.51 (16)
C5—C6—C7—O1	−8.2 (3)	C10—C9—C15—N1	−7.47 (19)
C1—C6—C7—O2	−9.5 (3)	C17—O4—C16—C9	−162.70 (17)
C5—C6—C7—O2	171.47 (17)	C8—C9—C16—O4	175.02 (15)
C7—O2—C8—C9	177.88 (14)	C15—C9—C16—O4	−56.9 (2)
O2—C8—C9—C16	68.2 (2)	C10—C9—C16—O4	58.2 (2)
O2—C8—C9—C15	−59.5 (2)	C16—O4—C17—O5	−6.0 (3)
O2—C8—C9—C10	−174.06 (14)	C16—O4—C17—C18	172.28 (17)
C16—C9—C10—O3	65.9 (3)	O5—C17—C18—C19	179.3 (2)
C8—C9—C10—O3	−53.1 (3)	O4—C17—C18—C19	1.1 (3)
C15—C9—C10—O3	−173.7 (2)	O5—C17—C18—C23	2.0 (3)
C16—C9—C10—C11	−113.60 (18)	O4—C17—C18—C23	−176.29 (19)
C8—C9—C10—C11	127.42 (17)	C23—C18—C19—C20	0.6 (3)
C15—C9—C10—C11	6.8 (2)	C17—C18—C19—C20	−176.7 (2)
C14—N1—C11—C12	0.5 (2)	C18—C19—C20—C21	0.3 (4)
C15—N1—C11—C12	178.82 (17)	C19—C20—C21—C22	−0.6 (4)
C14—N1—C11—C10	179.85 (17)	C20—C21—C22—C23	−0.1 (4)
C15—N1—C11—C10	−1.9 (2)	C21—C22—C23—C18	1.1 (4)
O3—C10—C11—N1	177.2 (2)	C19—C18—C23—C22	−1.3 (3)
C9—C10—C11—N1	−3.3 (2)	C17—C18—C23—C22	176.1 (2)
O3—C10—C11—C12	−3.9 (5)		

### Hydrogen-bond geometry (Å, °)

**Table:**

*D*—H···*A*	*D*—H	H···*A*	*D*···*A*	*D*—H···*A*
C4—H4A···O3^i^	0.93	2.59	3.299 (3)	133
C20—H20A···O3^ii^	0.93	2.44	3.269 (3)	149

Symmetry codes: (i) *x*, *y*, *z*−1; (ii) *x*+1, *y*, *z*.
